# LncRNA PFL contributes to cardiac fibrosis by acting as a competing endogenous RNA of let-7d: Erratum

**DOI:** 10.7150/thno.58328

**Published:** 2021-02-09

**Authors:** Haihai Liang, Zhenwei Pan, Xiaoguang Zhao, Li Liu, Jian Sun, Xiaomin Su, Chaoqian Xu, Yuhong Zhou, Dandan Zhao, Bozhi Xu, Xuelian Li, Baofeng Yang, Yanjie Lu, Hongli Shan

**Affiliations:** 1 Department of Pharmacology (State-Province Key Laboratories of Biomedicine-Pharmaceutics of China, Key Laboratory of Cardiovascular Research, Ministry of Education), College of Pharmacy, Harbin Medical University, Harbin, Heilongjiang 150081, P. R. China; 2 Northern Translational Medicine Research and Cooperation Center, Heilongjiang Academy of Medical Sciences, Harbin Medical University, Harbin, Heilongjiang 150081, P. R. China.

In our paper 1, the images of TGF-β1+let-7d groups in Figure 7I have misused that of the Control group. The correct version of the figure appears below.

The corrections made in this erratum do not affect the original conclusions. The authors apologize for any inconvenience or misunderstanding that this error may have caused.

## Figures and Tables

**Figure 7:**
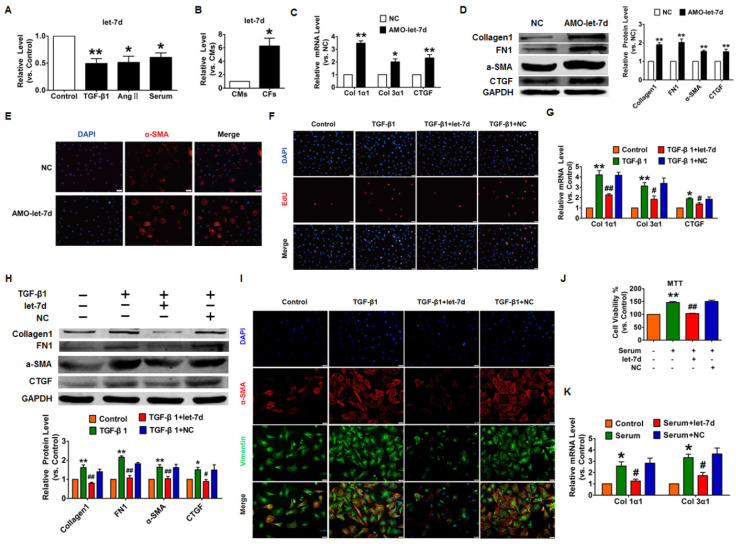
Overexpression of let-7d abrogated fibrogenesis in CFs. (A) Decreased expression of let-7d in CFs pretreated with TGF-β1, Ang II or serum. *p<0.05 and **p<0.01 vs. control; (B) Expression of let-7d in CMs and CFs. *p<0.05 vs. CMs. Inhibition of let-7d increased collagen 1α1 and collagen 3α1 mRNA expression (C) and promoted fibrogenesis (D) in CFs. *p<0.05 and **p<0.01 vs*.* NC. (E) Representative immunofluorescence images showing that suppression of let-7d promoted the transition of fibroblasts into myofibroblasts. Overexpression of let-7d mitigated TGF-β1-induced cell proliferation (F), collagen production (G), fibrogenesis (H) and the fibroblast-myofibroblast transition (I). *p<0.05 and **p<0.01 vs. control; ^#^*p*<0.05 and ^##^*p*<0.01 vs*.* TGF-β1. Forced expression of let-7d alleviated 20% serum-driven proliferation (J) and fibrogenesis (K) in CFs. *p<0.05 and **p<0.01 vs. control; ^#^*p*<0.05 and ^##^*p*<0.01 vs*.* serum. n=5-6 independent cell cultures.
